# Laurdan Discerns Lipid Membrane Hydration and Cholesterol Content

**DOI:** 10.1021/acs.jpcb.3c00654

**Published:** 2023-04-06

**Authors:** Hanna Orlikowska-Rzeznik, Emilia Krok, Madhurima Chattopadhyay, Agnieszka Lester, Lukasz Piatkowski

**Affiliations:** Faculty of Materials Engineering and Technical Physics, Poznan University of Technology, Piotrowo 3, 61-138 Poznan, Poland

## Abstract

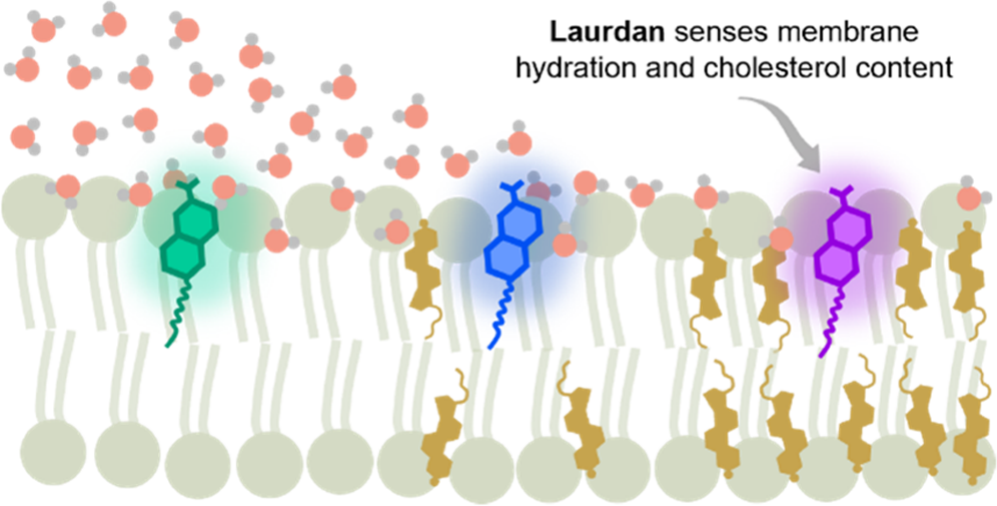

Studies of biological membrane heterogeneity particularly benefit from the use of the environment-sensitive fluorescent probe Laurdan, for which shifts in the emission, produced by any stimulus (e.g., fluidity variations), are ascribed to alterations in hydration near the fluorophore. Ironically, no direct measure of the influence of the membrane hydration level on Laurdan spectra has been available. To address this, we investigated the fluorescence spectrum of Laurdan embedded in solid-supported lipid bilayers as a function of hydration and compared it with the effect of cholesterol—a major membrane fluidity regulator. The effects are illusively similar, and hence the results obtained with this probe should be interpreted with caution. The dominant phenomenon governing the changes in the spectrum is the hindrance of the lipid internal dynamics. Furthermore, we unveiled the intriguing mechanism of dehydration-induced redistribution of cholesterol between domains in the phase-separated membrane, which reflects yet another regulatory function of cholesterol.

## Introduction

Water hydrating biological membranes are unequivocally essential for the maintenance of cell viability. Living in peculiar physicochemical cooperation, water stabilizes the structure and dynamics of the lipid bilayer^1^ and mediates its interactions with other biomolecules,^2^ while lipids affect the spatial arrangement and dynamics of adjacent water molecules.^3^ It is generally accepted that biomembranes exist in a fully hydrated environment; however, it should be noted that cell life also involves the local, temporary membrane dehydration events, such as adsorption of biomacromolecules or lipid bilayer fusion, the latter being a key phenomenon to subcellular compartmentalization, cell growth, neurotransmission, fertilization, viral entry, or exocytosis.^4,5^ Hence, it is clear that the mechanistic understanding of such events requires detailed insights into the local membrane hydration state. Yet, its determination is nontrivial, since the extent to which water interacts with different segments of the lipid bilayer is modulated by various factors such as temperature, the type of lipid headgroup, acyl chain composition, and the phase state of a lipid bilayer.^6,7^ Membrane phase is largely governed by cholesterol (Chol) content, which is a key regulator of acyl chains' conformational order and lipid dynamics.^8^ Pure phospholipid bilayers are known to exist either in the solid (gel) or liquid-disordered (L_d_) phase. At sufficient concentration, cholesterol promotes the formation of the intermediate phase known as the liquid-ordered (L_o_) phase, which may coexist with the other two.^9^ The L_o_/L_d_ coexistence, manifested as lateral heterogeneity on a nanometer and micrometer scale, is considered to be the most relevant from the biological perspective.^10,11^ One of the approaches to assess membrane heterogeneity is to employ a fluorescent environmentally sensitive probe immersed in a bilayer, such as the most commonly used Laurdan.^12^ Upon electronic excitation, the Laurdan dipole moment significantly increases, giving rise to dipolar relaxation of the surrounding molecules. The rearrangement of the immediate environment consumes the energy of the excited Laurdan molecule, manifested as a red shift of the emission spectrum.^13^ This accounts for the extreme sensitivity of Laurdan to the polarity and rate of dipolar relaxation of its immediate environment. In the literature, Laurdan is used to probe the membrane heterogeneity referring, often interchangeably, to different membrane physicochemical properties, including lipid order,^14^ hydration,^15^ or the general term fluidity,^16^ and although these features are related to each other, they are not equivalent. Nevertheless, regardless of the property, any shift in the emission spectrum has been taken as a consequence of alterations in the number and/or mobility of water molecules near Laurdan’s fluorescent moiety, below the glycerol backbone of the phospholipids. Ironically, despite widespread use for more than four decades, no direct measure of the influence of the membrane hydration state on Laurdan spectra has been available.

Here, we investigated, for the first time, the spectral response of Laurdan to dehydration of biomimetic cell membranes, directly compared it with the effect of increasing cholesterol content, and elucidated the molecular mechanisms that govern the observed changes. By monitoring the fluorescence spectral characteristics of Laurdan during dehydration of the membrane with L_o_/L_d_ coexistence, we unveiled an intriguing mechanism of interphase cholesterol redistribution, that is of relevance for membrane-centered cellular events. Our results have important implications for the proper interpretation of data obtained with this and other environmental probes, especially when assessing membrane heterogeneity in living systems, where numerous effects, including local variations in hydration and cholesterol content, can be encountered, often simultaneously.

## Methods

### Materials

Lipids 1,2-dimyristoleoyl-glycero-3-phosphocholine (di14:1-Δ9*cis*-PC), egg yolk sphingomyelin (eggSM), cholesterol (Chol), 1,2-dipalmitoyl-*sn*-glycero-3-phosphocholine (DPPC), and 23-(dipyrrometheneborondifluoride)-24-norcholesterol (TopFluor-Chol) were supplied by Avanti Polar Lipids (Alabaster, AL). Fluorescent probe 6-dodecanoyl-2-dimethylaminonaphthalene (Laurdan), phospholipid 1,2-dioleoyl-*sn*-glycero-3-phosphoethanolamine labeled with Atto 633 (Atto 633-DOPE), monosialoganglioside (GM1) from bovine brain, and chloroform (HPLC grade) were purchased from Merck KGaA (Darmstadt, Germany). Alexa Fluor 594 conjugated with cholera toxin subunit B (Alexa Fluor 594-CTxB) was obtained from Molecular Probes, Life Technologies (Grand Island, NY). Buffer reagent 4-(2-hydroxyethyl)piperazine-1-ethanesulfonic acid (HEPES PUFFERAN) was obtained from Carl Roth GmbH + Co., KG (Karlsruhe, Germany). Calcium chloride (CaCl_2_) was purchased from Chempur (Piekary Slaskie, Poland). Sodium chloride (NaCl) was supplied by PPH STANLAB Sp. z o.o. (Lublin, Poland). All compounds were used without further purification. Ultrapure water was acquired using the Milli-Q Direct Water Purification System from Merck KGaA (Darmstadt, Germany). Optical adhesive UV-activated glue Norland 68 was purchased from Thorlabs Sweden AB (Mölndal, Sweden). The sheets of mica used for the preparation of solid supports for the lipid membranes were obtained from Shree GR Exports Private Limited (Kolkata, India). Glass coverslips No. 0 were purchased from Paul Marienfeld GmbH & Co., KG (Lauda-Königshofen, Germany).

### Solid-Supported Lipid Bilayers Fabrication

Solid-supported lipid bilayers (SLBs) were prepared using vesicle deposition on a solid substrate procedure as described previously^1^ with appropriate modifications. SLBs of different compositions were examined: (i) pure di14:1-Δ9*cis*-PC, (ii) binary mixtures di14:1-Δ9*cis*-PC/Chol with varying Chol molar ratio (*x*_Chol_ = 0.1; 0.2; 0.25; 0.3; 0.4; 0.5; 0.6), (iii) equimolar ternary mixture di14:1-Δ9*cis*-PC/Chol/eggSM, (iv) pure DPPC, and (v) binary mixtures di14:1-Δ9*cis*-PC/DPPC with two DPPC molar ratios (*x*_DPPC_ = 0.1; 0.9). First, the membrane components were mixed along with the fluorescent probe(s) to form a chloroform solution of the specified composition with a final lipid concentration of 10 mM. The lipid to each fluorescent probe molar ratio was 1000:1. For the fluorescence spectral measurements, two probes—Laurdan and Atto 633-DOPE—were used, whereas, for the confocal microscopy experiments three probes—Atto 633-DOPE, TopFluor-Chol, and Alexa Fluor 594-CTxB-GM1 complex—were used to label the membrane. The appropriate solution was then dried under nitrogen gas, followed by desiccation in the vacuum chamber for at least 2 h. The lipid film was then hydrated in buffer solution (10 mM HEPES and 150 mM NaCl, pH adjusted to 7.4) to obtain a 10 mM lipid concentration. The lipid suspension was subjected to four cycles of heating to 60°C and vortexing, with each heating and vortexing step taking 1 min, producing multilamellar vesicles (MLVs). The lipid mixture was diluted 10-fold in a buffer to yield a 1 mM MLV suspension, and then distributed into sterilized glass vials and stored at −20°C for further use. The aliquoted MLV suspension of the desired composition was bath-sonicated for at least 10 min until the solution became transparent, indicating the formation of small unilamellar vesicles (SUVs). To prepare a solid support for SUV deposition, a small amount of immersion oil was deposited onto glass coverslip No. 0, over which a thin sheet of freshly cleaved mica, cut beforehand as round plates with a diameter of 9.53 mm (3/8 inches), was placed and adhered with UV-activated glue around the periphery of the substrate. A microcentrifuge tube’s lid and bottom were cut off and the resulting cylinder was placed on a coverslip and sealed with silicone to form a reservoir with mica at the bottom. 100 μL of SUVs suspension was deposited on the mica surface followed by the immediate addition of 2 μL of 0.1 M CaCl_2_ solution. After 30 s, 600 μL of the previously used buffer solution was added to prevent the hydration layer from drying out. After 30 min of incubation at ambient temperature, the SLB was rinsed 10 times with 2 mL of buffer solution to wash out the excess, unburst vesicles. Finally, the remaining volume of the tube was filled with the buffer solution, and this condition is called the fully hydrated state of the membrane throughout the paper.

### SLB Hydration-State Control

To perform a direct measurement of the effect of the hydration state of the lipid bilayer on the Laurdan fluorescence spectrum, we employed our home-built humidity control setup,^1,17^ assuring a controlled drying process with a slow and sequential decrease in the relative humidity (RH) of the membrane environment. The setup consists of a nitrogen gas (N_2_) cylinder, three flow meters, three manual valves, a reservoir with water (for water-vapor saturation), and an electronic hygrometer. In brief, to reduce the SLB hydration, bulk water was first removed with a micropipette from the sample container until no buffer droplets on the mica surface were visible to the naked eye. Nitrogen gas of 95% RH was then immediately, and gently blown into the sample container. The relative humidity of N_2_ was regulated by mixing wet (water-vapor-saturated, 95% RH) and dry (0% RH) gas streams. Wet and dry N_2_ gas flows were individually regulated by two manual valves while monitoring the readings of two flow meters connected to the flow paths. A third flowmeter and manual valve were used to keep the final N_2_ gas flow rate constant at ∼1.2 L/min throughout the experiment. The electronic hygrometer allowed monitoring of the relative humidity and temperature of the final gas flow, indicating the possible need for adjustment. The dehydration was performed from 95 to 80% RH and further in steps of ∼10 to 0% RH. The SLB atmosphere was equilibrated to the specified relative humidity after about 10 min, and only then were the sample imaged and Laurdan emission spectra recorded.

### SLB Imaging and Steady-State Emission Spectra Acquisition

The main experiments were carried out on a manual, inverted microscope (Carl Zeiss, Axiovert 200). The excitation beam at 370 nm was provided by a pulsed supercontinuum laser (NKT Photonics, SuperK FIANIUM FIU-15) equipped with a UV extension unit (NKT Photonics, SuperK EXTEND-UV). In all of the experiments described, we used nonpolarized excitation. A 50/50 beam splitter was used to reflect the excitation light into an oil immersion objective (Carl Zeiss, EC Plan-Neofluar 40*x*/1.30), which focused the beam to a diffraction-limited spot in the sample plane. The epifluorescence signal was spectrally filtered using a 380 nm long-pass filter (Semrock, FF01-380/LP-25) and guided to a single photon counting module (Hamamatsu Photonics, C11202-100) for imaging purposes or to a spectrograph (Andor, Kymera 328I-C), where it was spectrally dispersed with a 150 lines/mm grating and subsequently detected with an electron multiplying charge-coupled device camera (Andor, iXon 888 UCS-BB), precooled to −70°C, for spectral measurements. One or the other detection path was selected with the help of a remotely controlled mirror. Single photon counting module counts were read and converted to a digital signal by data acquisition card (National Instruments Corporation, NI USB-6363). The sample was scanned across the fixed laser foci with a piezoelectric nanopositioning stage (Mad City Labs, Nano-LPS200) in the *x*–*y* dimension. Nano-Drive 3 controller (Mad City Labs) was used for controlling the scanning stage. Image reconstruction and positioning of the sample were controlled using a home-made LabVIEW program. To avoid excessive photobleaching, sample illumination was synchronized with data acquisition using an optical beam shutter (Thorlabs, SHB1T).

Monitoring of the cholesterol distribution in the SLB with multiple probes as a function of membrane hydration was realized using a laser-scanning confocal upright microscope (Carl Zeiss, LSM 710) with an oil immersion objective (Carl Zeiss, EC Plan-Neofluar 40*x*/1.30). Lasers of wavelengths 633, 488, and 543 nm were used for the excitation of Atto 633-DOPE, TopFluor-Chol, and Alexa Fluor 594-CTxB-GM1, respectively. The laser power was adjusted during imaging to avoid excessive photobleaching of the sample.

### Fluorescence Spectra Analysis

Laurdan generalized polarization (GP) was calculated from the equation introduced by Parasassi et al.^18^ and most commonly used in the literature: , where *I*_440_ and *I*_490_ are fluorescence intensities averaged over five data points (∼2 nm) around 440 and 490 nm, respectively. The averaging was done to compensate for the noise present in the spectra. Each GP value demonstrated in the figures is averaged over at least 10 different spots from each of the samples at a particular membrane hydration state or each cholesterol molar fraction (the number of samples varies from experiment to experiment and ranges from 1 to 4). The uncertainties were calculated as standard deviations.

Spectral decomposition of the fluorescence spectra acquired for the samples with specific composition and at specific conditions was done using two log-normal functions in the form^19^
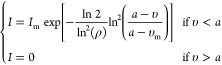
1where *I* represents the fluorescence emission intensity, *I*_m_ is the maximum of intensity, υ is the wavenumber, υ_m_ is the spectral position of the maximum intensity of the log-normal function,  is the asymmetry of the function (υ_max_ and υ_min_ represent the wavenumber values at half intensity), and *a* is the limiting wavenumber: . First, fluorescence spectra (averaged from at least five spots from each of the samples at a particular membrane hydration state or each cholesterol molar fraction) were fitted to a sum of two log-normal functions, independently for each sample and for each hydration/cholesterol content. In the fitting procedure, performed in Matlab, emission intensity *I*_m_, spectral position of the maximum intensity of the log-normal function υ_m_, as well as spectral positions determining the asymmetry of the function υ_max_ and υ_min_ were all kept as free parameters. The values for all of the parameters were restricted to take up physically meaningful values. To ensure that the two log-normal functions do not exhibit excessive asymmetry, we restricted υ_m_ – υ_min_ to take up values no larger than 1.5 times υ_max_ – υ_m_. All fitted parameters took values within the imposed bounds for over 90% of the fitted spectra. We point out that throughout the manuscript and the Supporting Information, all spectral data are presented in the wavelength space—experimental data are acquired in the wavelength space—thus, such a representation is more intuitive and also can be easily compared with other literature data showing Laurdan fluorescence spectra.

From the individual spectral fits, it was evident that the two log-normal functions (referred to as short-wavelength and long-wavelength bands in the main text) describe all of the acquired spectra very well (see Figure S1a,b), yielding high values of the coefficient of determination (*R*^2^ > 0.993 for all fitted spectra). Moreover, we note that the spectral position of the maximum intensity (υ_m_) of each fitted function did not exhibit significant changes as a function of hydration/cholesterol content, clearly pointing at the interconversion of the two (short wavelength/long wavelength) populations (see Figure S1c,d). The observed frequency shifts of the two bands for all hydration/cholesterol conditions are small (∼5–10 cm^–1^) with respect to the separation between the bands (>50 cm^–1^) and are random rather than showing a specific trend.

Next, we performed global fits (*n* spectra for all hydration conditions or cholesterol content for each sample with the specific composition) in which υ_m_ was kept as a global parameter for each of the two bands. All other parameters were allowed to vary for each sample condition. We used averaged parameter values from individual fits as starting parameters for the global fit. An exemplary result of the global fit is shown in Figure S2.

The populations of Laurdan experiencing solvent relaxation and of Laurdan embedded in a nonrelaxing environment were obtained by integrating the short-wavelength and long-wavelength bands, respectively, and represented as band areas relative to the area of the entire fluorescence spectrum [%].

It should be emphasized that dehydration of the lipid membrane does not affect its integrity and does not lead to the introduction of structural defects. The collected fluorescence emission spectra are highly reproducible (see Note 1 in the Supporting Information), both within the same sample as well as between different samples. Typically, 10–30 emission spectra from distinct spots were measured at each hydration condition for each sample and, importantly, the minute differences (mainly in absolute intensity) are much smaller (see Figure S3) than the differences between emission spectra for different hydration states.

## Results and Discussion

**Figure 1:**
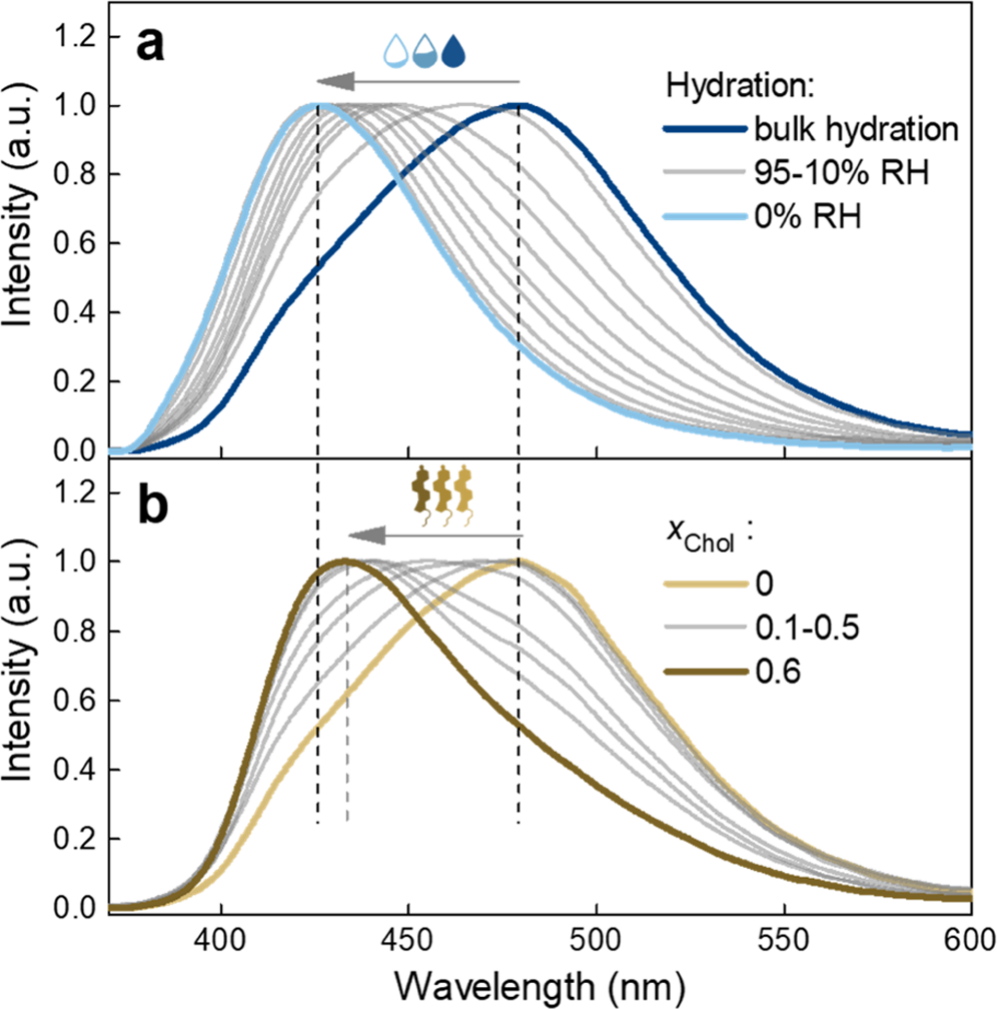
Changes in the fluorescence spectrum of Laurdan embedded in the di14:1-Δ9*cis*-PC SLB induced by (a) decreasing hydration state from bulk hydration down to 0% RH of the atmosphere surrounding the membrane (the intermediate hydrations are 95, 80, 70, 60, 50, 40, 30, 20, and 10% RH) and (b) increasing cholesterol molar fraction *x*_Chol_ from 0 up to 0.6 (the intermediate molar fractions are 0.1, 0.2, 0.25, 0.3, 0.4, and 0.5). Laurdan emission spectrum for each hydration step and each *x*_Chol_ is averaged over two different samples, smoothed using a fast Fourier transform filter, and normalized.

Changes in the steady-state fluorescence spectrum of Laurdan embedded in the solid-supported lipid bilayers (SLBs) composed of a pure phospholipid (di14:1-Δ9*cis*-PC) resulting from membrane dehydration are depicted in Figure 1a. The membrane hydration state was varied by applying a drying process with a slow and sequential reduction in the sample environment’s relative humidity (RH).^1^

**Figure 2:**
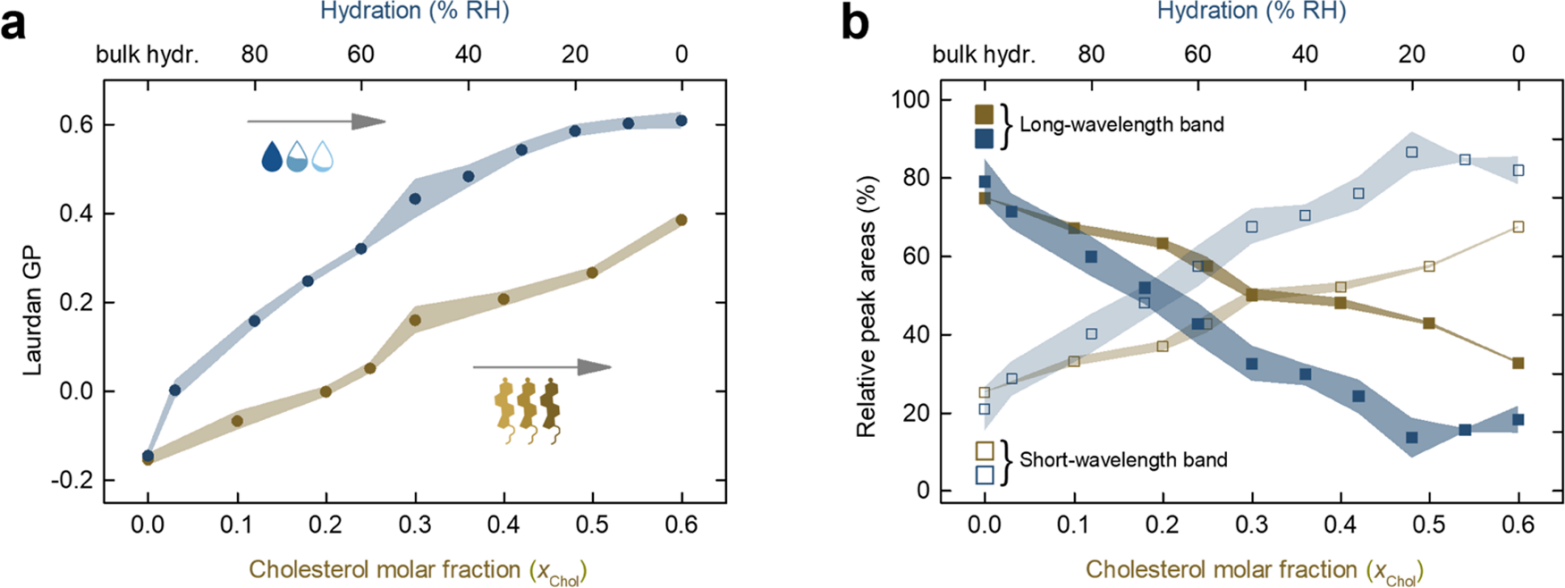
(a) Laurdan GP as a function of membrane hydration and cholesterol molar fraction in a di14:1-Δ9*cis*-PC SLB. (b) Relative area of the two log-normal components that give the best fit to the Laurdan emission spectra in the same membrane system. The uncertainties are standard deviations, denoted as shadows around mean values.

The fluorescence spectrum of Laurdan in the fully hydrated SLBs is characterized by a broad band with its maximum centered at ∼480 nm, a value that is typically attributed to the L_d_ phase,^20^ congruent with the report that at room temperature di14:1-Δ9*cis*-PC lipids form the disordered phase.^21^ As the hydration decreases, the spectrum exhibits a progressive blue shift. After drastic dehydration (0% RH), the probe’s emission spectrum resembles that characteristic of ordered membranes (in the gel phase) with the maximum centered at ∼430 nm.^22^ Consequently, the observed changes are reflected in the Laurdan generalized polarization (GP), which is a commonly used parameter to assess the overall membrane order (Figure 2a, blue part).^19^ It is defined as GP = (*I*_440_*– I*_490_)/(*I*_440_ + *I*_490_), where *I*_440_ and *I*_490_ are the fluorescence emission intensities at 440 and 490 nm, respectively.^18^

Theoretically, GP can assume values between 1 and −1; however, in the lipid membranes, it does not reach its extreme values and typically scales from 0.6 (the most ordered) to below −0.1 (the least ordered).^23^ As can be seen from Figure 2a (blue part), for a fully hydrated membrane, GP has a negative value, indicative of a disordered bilayer. Upon removal of bulk water and equilibrating the membrane with an atmosphere of 95% RH, the GP takes on a value close to zero and gradually increases with a further reduction of water content, reaching a maximum of 0.62 ± 0.04 for a water-depleted membrane (0% RH), a characteristic of a gel phase. We note here that the collected fluorescence emission spectra are highly reproducible, both when acquired within the same sample from multiple spots as well as between different samples, and that the minute spectral variation is much smaller than the differences between emission spectra for different hydration states (see Note 1 in the Supporting Information and Figure S3).

Interestingly, we verified whether pure Laurdan deposited on a solid support spectrally responds to changes in hydration by exposing its dry layer to bulk water (see Note 2 in the Supporting Information and Figure S4). However, it neither shifts the spectrum nor changes its shape. We infer that water molecules do not permeate the dried tightly packed aggregates/crystals of Laurdan and that the intermolecular interactions between the probe’s molecules dominate over interactions with the interfacial water that would decrease the emitted energy by dipolar relaxation.

To confront the pure effect of membrane dehydration with the influence of cholesterol on the Laurdan response, we examined the membranes composed of a binary mixture of di14:1-Δ9*cis*-PC with varying molar fraction of cholesterol (*x*_Chol_). Changes in the probe’s emission spectrum resulting from the increasing *x*_Chol_ are depicted in Figure 1b. The emission peak is significantly affected by the sterol concentration, exhibiting the shift in the same direction as in the case of decreasing hydration. At first glance, changes due to membrane dehydration and due to addition of cholesterol appear very similar. Nevertheless, a few significant differences can be pointed out. Up to *x*_Chol_ = 0.2, the long-wavelength shoulder of the spectrum (∼490–550 nm) virtually does not change. Only at *x*_Chol_ = 0.25 and above is a progressive decrease in its intensity observed. In contrast, the lower wavelength part of the spectrum initially exhibits drastic shift and then stops changing above *x*_Chol_ = 0.3. These were not observed in the case of membrane dehydration, for which gradual change of the spectral position was observed throughout the entire dehydration trajectory. Another characteristic feature, evident when analyzing the change in the spectral shape with increasing *x*_Chol_, is a spectral shoulder (∼480 nm) that stands out over a wide range of cholesterol concentrations. Most importantly, comparing the extent of the blue shift, it is apparent that membrane dehydration down to 0% RH causes greater changes than the highest cholesterol content, *x*_Chol_ = 0.6. The same conclusions can be derived by analyzing the course of the GP parameter (Figure 2a, brownish part). The increase in cholesterol is accompanied by an increase in Laurdan GP, but to a much lesser extent compared to the pure effect of water depletion, as it reaches a maximum of 0.38 ± 0.03 for the highest *x*_Chol_. We also verified higher molar fractions of cholesterol (0.7 and 0.8), but as expected, they did not produce further changes in the fluorescence spectrum, which is reasonable given the limited solubility of cholesterol in the phospholipid membrane.^24^ To evaluate whether the other ordering lipid, such as the saturated DPPC, produces a similar spectral response of the probe, we measured Laurdan’s fluorescence spectrum in SLBs composed of pure DPPC as well as mixtures of DPPC with di14:1-Δ9*cis*-PC at molar fractions of DPPC equal to 0.1 and 0.9. Intermediate molar fractions were omitted to avoid phase separation.^25^ Data are presented in Figure S5 and discussed in Supporting Information Note 3. In a nutshell, at a very low molar fraction *x*_DPPC_ = 0.1, DPPC has a comparable effect on Laurdan’s fluorescence spectrum to cholesterol at *x*_Chol_ = 0.1. However, when higher molar fractions of these molecules are considered, it is clear that cholesterol has a stronger effect on Laurdan’s spectral response than DPPC, which highlights the unique character of this sterol molecule.

It can be noted that the Laurdan fluorescence spectrum has a complex line shape, indicative of a heterogeneous local environment. Although due to the dynamic nature of the phospholipid bilayer with existing packing defects,^26^ and the nonuniform, to some extent, insertion depth^27^ and orientation^28^ of Laurdan in the membrane, there may be different subpopulations of Laurdan molecules experiencing distinct environments, we consider that the ability of the local environment to adapt to the excited Laurdan molecule (dipolar relaxation) is the major determinant of Laurdan’s fluorescence spectral properties. In the simplest approach, the Laurdan emission can be modeled in terms of a simple two-state model, assuming that the steady-state spectrum contains two contributions, a short-wavelength band reflecting Laurdan population experiencing little or no dipolar relaxation and a long-wavelength band associated with Laurdan within the readily relaxing environment. Thus, to gain further insight into the probe’s local molecular environment upon membrane dehydration and addition of cholesterol, we performed spectra decomposition using two log-normal line shapes as proposed by Bacalum et al.^19^ (Figure S2 and Methods Section). The results of such an analysis are plotted in Figure 2b as the relative areas of the short-wavelength and long-wavelength bands, reflecting the percentages of Laurdan populations associated with the nonrelaxed and relaxed local solvent environment, respectively, as a function of the membrane hydration (blue part) and cholesterol content (brownish part). The exemplary extracted spectra for different cholesterol molar fractions are shown in Figure S2.

The Laurdan fluorescence spectra, at all degrees of hydration, can be well described by a superposition of two log-normal line shapes, with peaks around 475 and 427 nm, confirming that the fluorescence decay is mainly due to transitions from only two different excited energy levels. In a pure phospholipid bilayer under fully hydrated conditions, most (∼79%) of the fluorescence emission is due to the long-wavelength transition of Laurdan residing in hydrated, relaxed environment (Figure 2b, blue part). As water content is lowered, the population of Laurdan molecules that experiences a dipolar relaxation starts decreasing, giving rise to the short-wavelength transition. A steady decrease of the population within a relaxed environment (and a concomitant increase of the population experiencing the nonrelaxed medium) is observed from a fully hydrated state to around 20% RH. Below this value, the band fractions reach a plateau with the relative populations of the two Laurdan populations accounting for ∼18 and ∼82%, respectively, and hence the opposite of full hydration.

Noticeably, cholesterol produces more subtle changes (Figure 2b, brownish part). Analogously to the dehydration process, the Laurdan fluorescence spectra, at all *x*_Chol_, can be well reconstructed by a superposition of two log-normal lines, with maxima around 482 and 430 nm (Figure S2). Throughout the analyzed range of cholesterol concentrations, the differences in the proportion of the populations emitting from within the relaxed and nonrelaxed solvent environments are not as pronounced compared to the effect of decreasing membrane hydration. In other words, compared to dehydration, even at high *x*_Chol_, a considerable number of Laurdan molecules experience a dipolar relaxation.

Now, let us consider the physical origin of the observed changes. Golfetto et al.^29^ presented an interesting approach combining the fluorescence lifetime detection and phasor analysis and showed for Laurdan in solution and Laurdan embedded in model and life cell membranes the ability to disentangle the effects of the extent of hydration versus cholesterol content. However, it should be noted that in this work, the membrane hydration level has not been altered directly. The only variables that were controlled were cholesterol content (in both lipid vesicles and live cells) and epidermal growth factor stimulation (in the case of live cells), and none of them change the membrane hydration state directly and in a specific way. The observed shortening of Laurdan’s fluorescence lifetime resulting from collisional quenching has been assigned solely to the reduction of the number of water molecules around the probe without considering other effects. Indeed, the first thing that comes to mind, when observing the shift of the spectrum toward shorter wavelengths as the membrane is dehydrated, is the gradual reduction in the amount of water molecules around the fluorophore. It must be emphasized, however, that there is compelling evidence pointing that the rationale behind dehydration-induced Laurdan’s spectral response must be different.

1. First of all, the Laurdan fluorescent moiety localizes below the glycerol backbone of phospholipids, near the *sn-1* carbonyls,^30^ where water molecules are scarce and strongly bound via hydrogen bonds to the lipid carbonyl oxygen atoms.^31−33^ Naturally, Laurdan emission is mostly sensitive to the changes in its direct vicinity, rather than at the level of phosphates or even the more outer parts of the membrane. In the lipid membrane interphase region, beyond the carbonyls, water is distributed around the phosphate and choline groups. The carbonyl and phosphate regions involve on average six hydrogen-bonded water molecules.^31^ The choline moiety, on the other hand, due to the nonpolar character of methylenes, cannot form H-bonds with adjacent water molecules. Instead, it organizes the water molecules via weak electrostatic and van der Waals interactions so that they form a clathrate structure around it, containing, in the case of a zwitterionic phosphocholine lipid, about six water molecules.^34^ In total, these twelve water molecules are considered a first hydration shell. Subsequent hydration shells exclusively incorporate water molecules that are unbound to lipids and are assumed to be localized mostly in the outer parts of the membrane.^35^ During dehydration, it is the strength of intermolecular interactions that governs the order of desorption of water molecules. As such, loose water molecules interacting only with each other, through relatively weak hydrogen bonds, along with the water molecules directly associated with phospholipids via the weak van der Waals interactions are removed first. These are followed by desorption of water molecules bound more strongly to polar residues of phospholipids.^35^ It has been shown that upon removal of bulk water and exposing the lipid bilayers to 95% RH, the first solvation shell is largely preserved and only further reduction of hydration degree breaks it down.^1^ However, even extreme dehydration does not remove water strongly bound to lipids, particularly those associated with the carbonyls.^34^ Having this molecular picture in mind, we infer that upon membrane dehydration, the most drastic changes in hydration occur in the outer regions of the phospholipid bilayer, while the number of water molecules in the vicinity of Laurdan fluorophore barely changes. Thus, the rationale behind Laurdan's response and the change in the local dipolar relaxation properties cannot result solely from the reduction of the number of water molecules in the vicinity of Laurdan. This points toward changes in the kinetics of Laurdan’s local environment.
2. In fact, the nanosecond solvent relaxation kinetics, revealed by the time-dependent fluorescence shift measurements of Laurdan in phospholipid bilayers, is associated with the collective rearrangement of the hydrated *sn-1* carbonyls and not water molecules themselves.^36^ In addition, in the same work, it was demonstrated that GP calculated from the steady-state Laurdan emission spectra correlates well with rearrangement kinetics of the immediate vicinity of the fluorophore and not the total spectral shift (which mirrors the polarity and thus the number of water molecules).^36^ This implies that Laurdan GP primarily reflects the mobility of hydrated functional groups of lipids at the Laurdan level rather than the extent of water penetration.
3. The mobility of lipid carbonyls, in turn, has been found to be dependent on the local hydrogen bond network dynamics.^37^ Noteworthy, a number of experimental^38−40^ and molecular dynamics (MD) simulation^32^ studies unveiled the slowdown of interfacial water dynamics induced by membrane dehydration.^32,38^ These results indicate an increased residence time of bound water molecules within the lipid polar groups as the water content decreases. The more persistent the hydrogen bonds between water molecules and carbonyl oxygens, the more restricted dynamics of hydrated carbonyls, and consequently, the lower ability of these oscillators to adapt to the excited state of Laurdan.
4. In addition to the slowdown of interfacial water dynamics, both the structural and dynamical properties of lipid bilayers have been found to be affected by the water content.^1,38,41,42^ Membrane dehydration results in a decrease in the area and volume per lipid and a concomitant increase in membrane thickness, as well as a slowdown in the lipid translational and rotational mobility, ultimately leading to a liquid-disordered to gel-phase transition.

Our results of the log-normal decomposition reinforce the idea that during membrane dehydration, there is no significant change in the hydration level at the Laurdan site. Had the number of water molecules aligning around the Laurdan dipole decreased, indicating a decrease in the polarity of the immediate vicinity of the fluorophore, a shift in the peak wavelength would have been observed. Instead, we obtained stable positions of the peaks (see Figure S1).

When interpreting the steady-state Laurdan emission spectrum, it is important to keep in mind that it is the resultant not only of the extent of dipolar relaxation but also of the interplay between its rate and the probe’s fluorescence lifetime. In other words, Laurdan emission can be red-shifted only if solvent dipolar realignment occurs while Laurdan is in its excited state. If the dipolar relaxation completes within Laurdan fluorescence lifetime, the steady-state spectrum captures the fully solvent-relaxed state. On the other hand, when the fluorescence occurs before the probe’s polar environment responds, the Laurdan steady-state spectrum appears as if dipolar relaxation has not occurred. It should be noted, however, that the main process that shortens the fluorescence lifetime of Laurdan is the collisional quenching by water molecules within the bilayer.^29^ Therefore, the excited-state lifetime is expected to be the shortest at fully hydrated conditions. Yet, at this hydration level, we observe a substantial red shift. Thus, while we do not assert that we capture the fully solvent-relaxed state, the emission occurs at least from a partially solvent-relaxed state. We assume that as the membrane hydration level decreases, Laurdan fluorescence lifetime does not change or at most increases.

Altogether, the above reasoning based on our observations as well as compelling evidence from previous studies show that the observed changes in the Laurdan fluorescence emission spectrum result from the hampered dipolar relaxation of Laurdans’ immediate environment. Therefore, we interpret the decrease in the area of the long-wavelength band as a diminishing population of Laurdan molecules for which the collective relaxation of the hydrated lipid groups completes within Laurdan’s fluorescence lifetime. In other words, as the water depletion in the bilayer progresses, the number of localized sites where hydrogen bond network and lipid dynamics allow for the hydrated carbonyls to reorient along the Laurdan excited-state dipole decreases.

The influence of membrane dehydration and the effect of cholesterol on the Laurdan emission spectrum are illusively similar, but not equivalent. Importantly, congruent with our spectral decomposition results, as suggested by Amaro et al.,^43^ based on the time-resolved emission spectra measurements, the presence of cholesterol does not significantly affect the polarity (number of water molecules) in the vicinity of the Laurdan fluorophore. Therefore, cholesterol-induced changes in the probe’s emission spectrum must also be associated with the reduced kinetics of dipolar relaxation. Intriguingly, both experimental and theoretical investigations revealed that contrary to dehydration, an increase in cholesterol content in the membrane leads to a rupturing of rigid interlipid H-bonds bridging two adjacent phospholipids, and an accompanying increase in the fraction of lipid–water H-bonds, which are faster and more mobile, thus overall leading to an enhancement in the water mobility at the interface.^44−46^ This effect would rather promote the dipolar relaxation around the Laurdan fluorescent moiety. On the other hand, cholesterol is known to induce phospholipid bilayer ordering, as manifested by a significant increase in the C–H bond order parameter of different segments in the acyl chains of lipids in nuclear magnetic resonance experiments.^47−49^ Interestingly, as reported by Warschawski and Devaux,^47^ the effect of cholesterol is much more pronounced than temperature or even the degree of unsaturation of the acyl chains. However, it should be emphasized that this relates only to the hydrophobic core of the membrane. The structural order parameters of the interfacial regions of the phospholipid bilayer, namely, the choline, phosphate, and glycerol backbone of the lipid headgroups along with the carbonyl region, remain virtually unaffected by the presence of cholesterol.^48,49^ Therefore, it is highly unlikely that it is the structural conformational ordering that causes such drastic changes in the Laurdan spectrum upon addition of cholesterol. It is worth noting, however, that the conformational order reflects the orientation of the C–H bond vector with respect to the bilayer normal averaged over the lipid ensemble and over time,^50^ but does not carry information about its dynamics. Importantly, recent work by Antila et al.^49^ unveiled that although cholesterol causes only marginal changes in the structural order of the membrane region where Laurdan resides, it significantly impedes the dynamics of the glycerol backbone and the associated carbonyls. This implies that the predominant phenomenon governing the cholesterol-induced blue shift of the Laurdan fluorescence spectrum is the slowing down of intralipid dynamics.

After determining both the effect of dehydration of a pure phospholipid bilayer and cholesterol incorporation, we evaluated the Laurdan response to dehydration of a phase-separated membrane, which is considered a much better mimic of biological membranes. To this end, we used an equimolar ternary mixture of di14:1-Δ9*cis*-PC, cholesterol, and egg sphingomyelin (eggSM), which at room temperature exhibits the L_o_/L_d_ phase coexistence (Figure S6). The use of SLBs as samples and fluorescence microscopy coupled with spectral detection enabled collection of spectra separately from the L_d_ and L_o_ domains. Under fully hydrated conditions, the emission of Laurdan in L_o_ is blue-shifted and significantly narrower than for Laurdan in the L_d_ phase (Figure S7), consistent with the previous observations.^51^ As expected, as the hydration level decreases, the fluorescence spectrum of Laurdan in L_d_ shifts toward shorter wavelengths (Figure S7a). Changes for L_o_ phase are much less pronounced (Figures S7b and S8); therefore, we focus here on the L_d_. The discussion on the insensitivity of Laurdan to dehydration of the L_o_ phase can be found in Supporting Information Note 4. Complete dehydration of L_d_ domains resulted in a smaller shift in the spectrum than dehydration of the single-component membrane, but greater than for a membrane with the maximum cholesterol content. It is worth noting that the lateral organization of the membrane was monitored between the spectra collection routes for distinct hydration states, and it was confirmed that the phase separation of the membrane remained virtually unaltered during the dehydration process (see Figure S6). Comprehensive data on this issue can be found in our previous work.^1^

**Figure 3:**
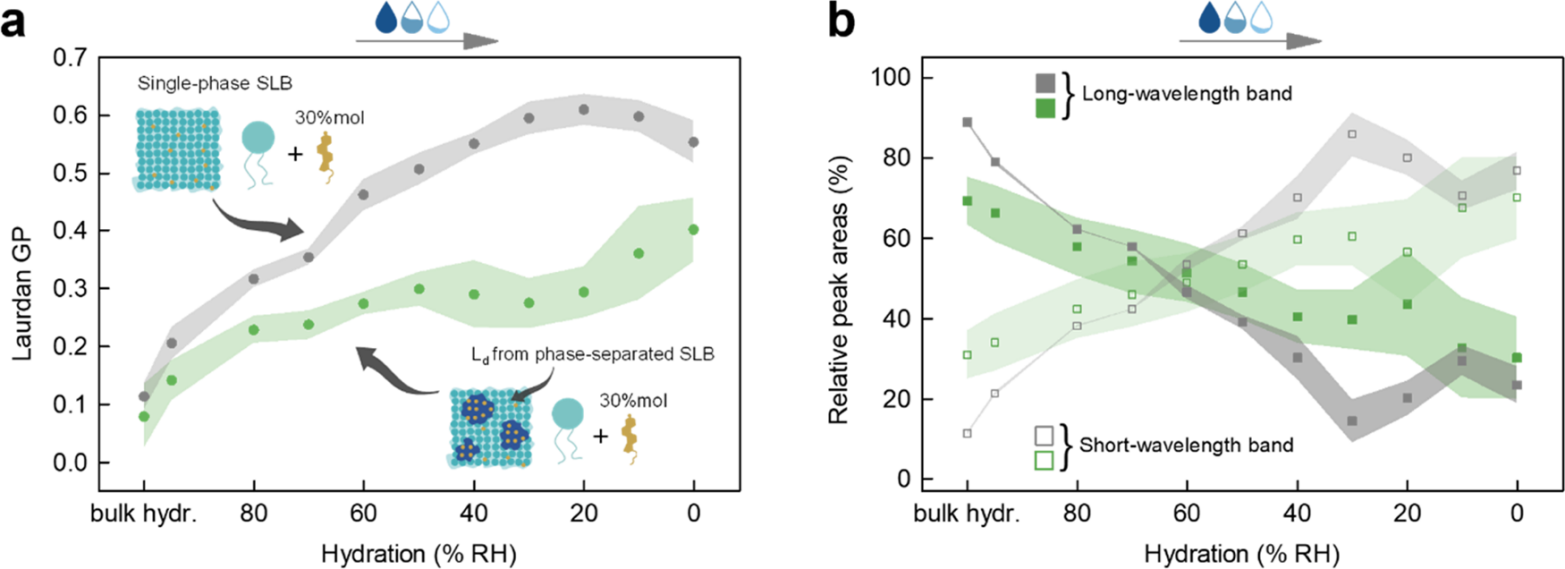
(a) Laurdan GP for L_d_ domains from phase-separated SLB composed of an equimolar mixture of di14:1-Δ9*cis*-PC/Chol/eggSM and for the counterpart from SLB without phase separation composed of a binary mixture of di14:1-Δ9*cis*-PC/Chol with *x*_Chol_ = 0.3 as a function of the hydration level. (b) Relative area of the two log-normal components that give the best fit of the Laurdan emission spectra in the same membrane systems as a function of hydration. The uncertainties are standard deviations, denoted as shadows around mean values.

Analysis of the GP parameter as a function of hydration level of L_d_ domains reveals an interesting behavior (Figure 3a, green part).

In the range from bulk hydration to 50% RH, a gradual, small increase in GP can be observed, after which it remains constant down to 20% RH, and then it increases slightly again. It is qualitatively different than in the case of pure phospholipid SLBs dehydration. Intrigued by this, we examined whether changes in GP caused by the dehydration process of a membrane without phase separation, but with the same composition as in the L_d_ domains, occur in the same way. By comparing the line shape of the Laurdan fluorescence spectra acquired from the L_d_ domains with the ones originating from the monophasic membranes with different *x*_Chol_, we inferred that under fully hydrated conditions, the *x*_Chol_ in L_d_ is in the range of ∼0.25–0.3. Therefore, to reproduce the molecular L_d_ composition in the membrane without phase separation, we prepared monophasic lipid membranes with *x*_Chol_ = 0.3 and yet again monitored Laurdan fluorescence during the dehydration process. It was assumed that since the two systems at bulk hydration are compositionally the same, the dehydration process would cause the same changes in Laurdan emission. Changes of the Laurdan spectrum in a membrane composed of a binary mixture of di14:1-Δ9*cis*-PC and Chol due to dehydration are demonstrated in Figure S9. The course of the GP value as a function of the hydration state of this membrane (Figure 3a, gray part) qualitatively resembles that for a pure phospholipid membrane (Figure 2a), except that it starts from a slightly higher value at full hydration (indicative of increased order) and stops changing below 30% RH but reaches the same average value of 0.62. But most importantly, and surprisingly, it does not resemble the trajectory for its counterpart from the phase-separated membrane (Figure 3a, green part). For the membrane without L_o_ domains present, the changes are steeper and do not exhibit a plateau in the range from 50 to 20% RH. In addition, in general, GP has significantly higher values for each of the hydration levels, except for bulk hydration, compared to the L_d_ domains from the phase-separated membrane. The spectral global analysis further highlights these differences (Figure 3b). The results are rather intriguing and indicative of an additional mechanism that counteracts and effectively softens the changes caused by dehydration of L_d_ in the phase-separated membrane. The observed trajectories (whether GP or resulting from spectral decomposition) resemble those observed for changing the cholesterol content (Figure 2b) rather than those for dehydration, suggesting that perhaps the cholesterol content in L_d_ phase changes. This is feasible as L_o_ phase contains more cholesterol (∼70% of all cholesterol in the membrane) and may act as a reservoir of cholesterol in the phase-separated membranes. Therefore, we reason that the peculiar dehydration-induced behavior of L_d_ phase in the phase-separated membrane might be due to the redistribution of components between L_o_ and L_d_ domains, most likely involving cholesterol. This would also rationalize our previous findings,^1^ that with reduced hydration, the hydrophobic mismatch between the L_d_ and L_o_ domains decreases significantly (cholesterol influx to L_d_ phase would increase its thickness, thus lessening the hydrophobic mismatch between domains). To test our hypothesis, we conducted fluorescence microscopy experiments with fluorescently labeled cholesterol, the results of which are depicted in Figure S10. Under fully hydrated conditions, as expected, higher intensity of TopFluor-Chol emission is found in L_o_ domains. However, with reduced hydration, the contrast diminishes until it becomes reversed, showing that the L_d_ domains contain more cholesterol. Therefore, it can be concluded that cholesterol influx from L_o_ to L_d_ phase counteracts the dehydration-induced extensive changes in fluidity. However, the study of cholesterol migration was not the aim of this work and an in-depth understanding and quantification of this phenomenon require additional experiments and analysis.

## Conclusions

In conclusion, we have provided a direct measure of the influence of the hydration level of the lipid bilayer on Laurdan spectra, which so far has been unattainable. We have shown that the effects of membrane dehydration and cholesterol incorporation on Laurdan’s fluorescence spectrum are illusively similar, and thus interpretation of data obtained with this probe should be done with caution. We evidence that the dehydration-induced changes in Laurdan’s emission spectrum result from the conformational ordering of lipids and hindrance of the lipid internal motions along with the slowdown of hydrogen bond network dynamics acting collectively to impede the dipolar relaxation around the probe’s excited-state dipole. In the case of cholesterol incorporation, for which neither hydrogen bond network relaxation slowdown nor static conformational ordering of the lipid bilayer region probed by Laurdan is observed, changes in the emission are likely caused only by the hampered dynamics of the glycerol backbone and the associated carbonyls, which rationalizes more subtle changes compared to membrane dehydration.

Moreover, by varying the composition and organization of the membranes (single-component, multicomponent phase-separated), we have shown that Laurdan’s spectral response to dehydration is much temperate in the presence of cholesterol. In other words, cholesterol to some extent counteracts the lowered relaxation properties of Laurdan’s local environment upon dehydration.

Furthermore, our unprecedented way to obtain biomimetic cell membranes with a well-controlled hydration state without interfering with membrane composition along with the detection of the Laurdan spectral response led us to unveil that the dehydration of the phase-separated membrane drives the redistribution of cholesterol between domains. It likely acts as a regulatory mechanism to prevent excessive deviations in fluidity that may destabilize the cell membrane and hence be harmful to the cell. This intriguing finding adds to the multiple actions of cholesterol toward the mechanochemical homeostasis of lipid membranes. Our results provide new insights at the intersection of physical chemistry, photo- and biophysics and should stimulate the design of a range of new experiments and simulations regarding the specificity and sensitivity of environmental probes.

## Supporting Information Available

Experimental results and information; additional results of the independent and global fitting procedure for different SLB systems, reproducibility of the measured spectra, fluorescence images and spectra of Laurdan crystals/aggregates on solid support in dry and wet conditions, fluorescence spectra of Laurdan embedded in lipid bilayers composed of pure DPPC and binary mixtures with di14:1-Δ9*cis*-PC, fluorescence microscopy images of a phase-separated SLB as a function of the membrane hydration state, fluorescence spectra of Laurdan in liquid-disordered and liquid-ordered domains from phase-separated SLB and in one-phase SLB with the molar fraction of cholesterol 0.3 as a function of the membrane hydration state, generalized polarization of Laurdan in the liquid-ordered phase as a function of membrane hydration, and confocal fluorescence microscopy images of a triple-labeled SLB composed of an equimolar mixture of di14:1-Δ9*cis*-PC, cholesterol, and egg sphingomyelin for three different membrane hydration states (PDF)
