# Supplementary material for: Laurdan Discerns Lipid Membrane Hydration and Cholesterol Content
Source: J Phys Chem B. 2023 Apr 6;127(15):3382–91. doi: 10.1021/acs.jpcb.3c00654 (PMC10123623; doi:10.1021/acs.jpcb.3c00654)
Supplement: Supplementary file 1 — jp3c00654_si_001.pdf [file jp3c00654_si_001.pdf]

Supporting Information

Laurdan Discerns Lipid Membrane Hydration and Cholesterol Content

Hanna Orlikowska-Rzeznik, Emilia Krok, Madhurima Chattopadhyay, Agnieszka Lester, Lukasz Piatkowski**

Faculty of Materials Engineering and Technical Physics, Poznan University of Technology, Piotrowo 3, 61-138 Poznan, Poland

*hanna.orlikowska@put.poznan.pl, tel. +48 61 665 31 99
*lukasz.j.piatkowski@put.poznan.pl, tel. +48 61 665 31 80

Supplementary Experimental Results and Information

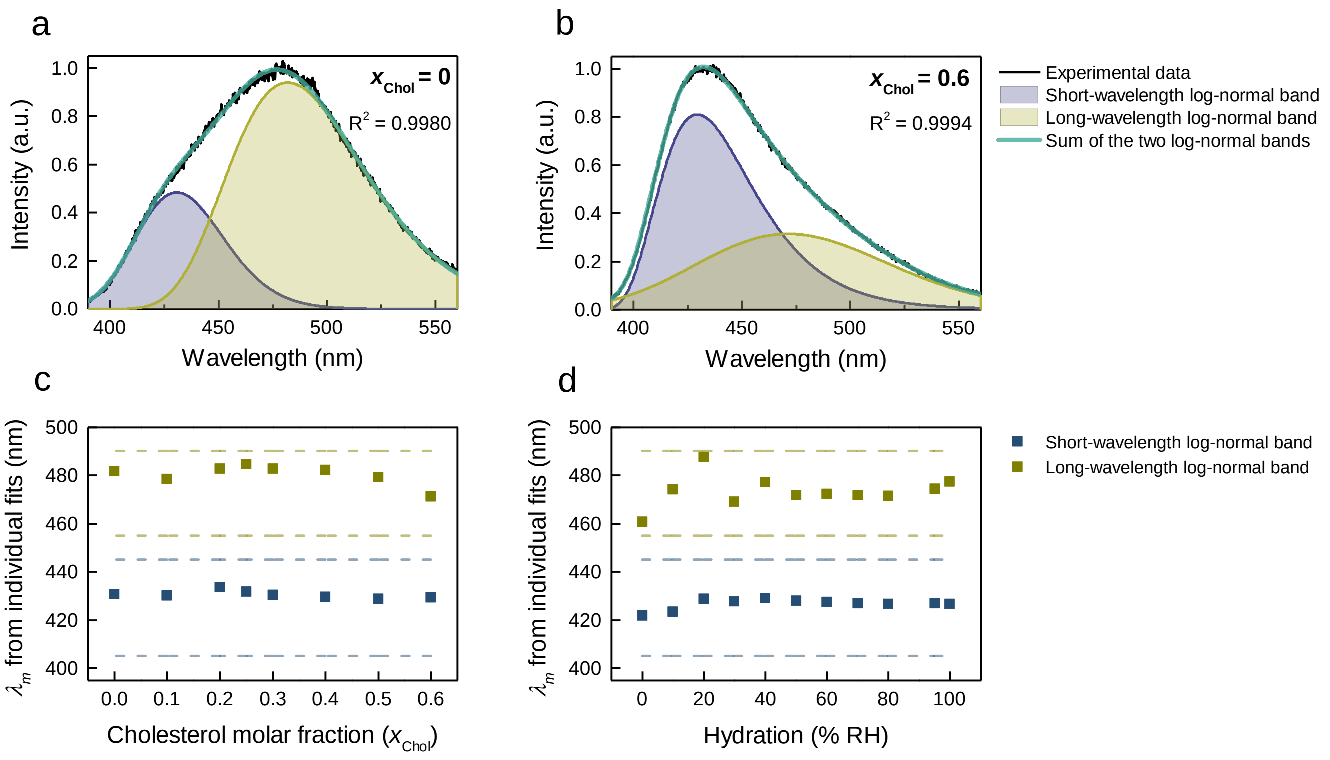

Figure S1. The exemplary results of the independent, individual spectra fitting procedure. Two-peak log-normal decomposition of the fluorescence spectra of Laurdan in an exemplary SLB composed of (a) pure di14:1-*Δ9cis*-PC and (b) the binary mixture of di14:1-*Δ9cis*-PC/Chol at x_{Chol} = 0.6 under fully hydrated conditions. Coefficients of determination R^2 are indicated in the figures. Spectral positions of the maximum intensity λ_m of the two log-normal functions as a function of (c) x_{Chol} for an exemplary single-phase SLB and (d) membrane hydration of the liquid-disordered phase for an exemplary phase-separated SLB. Blue and olive dashed lines correspond to the limiting range in the fitting procedure for short-wavelength and long-wavelength bands, respectively.

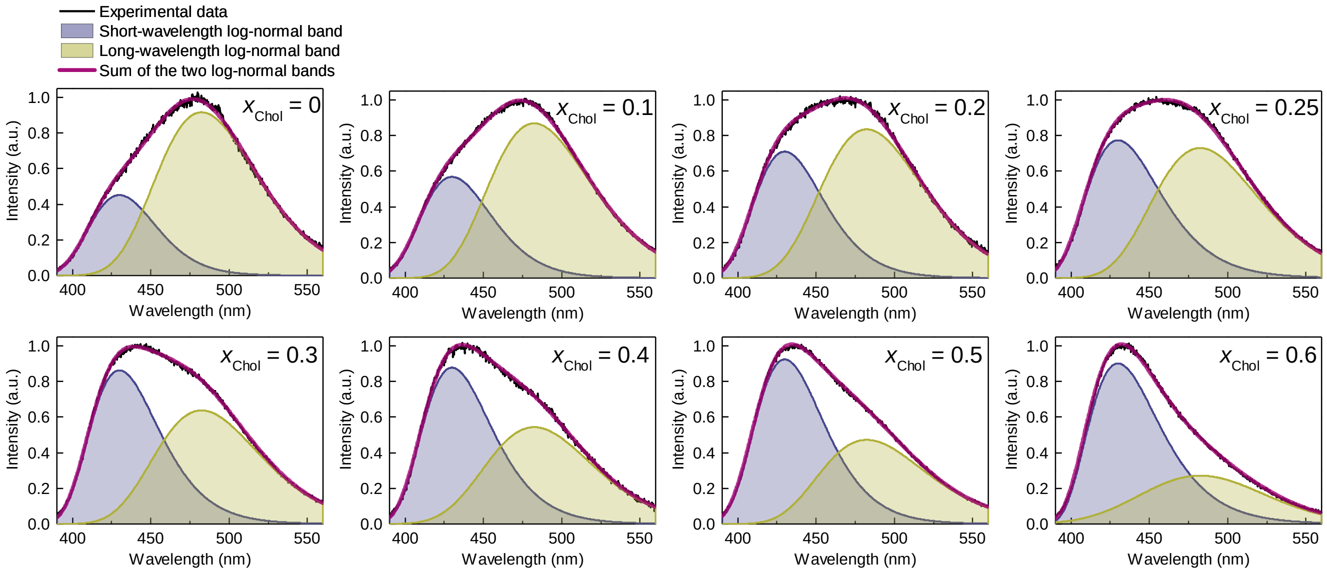

Figure S2. An exemplary result of the global fitting procedure. Two-peak log-normal decomposition of the fluorescence spectra of Laurdan in an exemplary SLB composed of di14:1-*Δ9cis*-PC and different cholesterol molar ratios $x_{Chol.}$ Coefficient of determination $R^2 = 0.9990$.

Note 1.

The collected fluorescence emission spectra are highly reproducible, both when acquired within the same sample as well as between different samples. Typically, 10 to 30 emission spectra from distinct spots, separated by a distance ranging from a few to thousands micrometers (the diameter of the mica flake is 8 mm), were measured for each membrane hydration state. As an example, in figure S1 we show 20 spectra collected at 50% RH during the dehydration and 10 during the rehydration process. First of all, spectra for a 50% RH for a given de(re)hydration process are just on top of each other, indicating negligible variability of the Laurdan environment within the sample. Secondly, changes in the spectrum are fully reversible, exhibiting only small hysteresis, resulting most probably from the inaccuracy of our humidity control system. Most importantly, the minute differences (mainly in absolute intensity) are much smaller than the differences between emission spectra for different hydration states.

Moreover, it should be emphasized that the used dehydration methodology leads to the preservation of membrane structure upon dehydration. We devoted a significant part of our previous work[1] to this topic and we are confident that, except for the deposition of a few aggregates on top of the membrane, the lipid bilayer integrity upon dehydration is not affected, provided that the membrane has been subjected to a well-controlled, gradual decrease in hydration level. Confocal images of solid-supported lipid bilayers (SLBs) labeled with multiple fluorescence probes, together with our most recent AFM studies (data not published) indicate that no significant changes occur in the quality of the phase-separated membrane structure (both in micro- as well as nano-scale, in the quality of the phase-separated membrane structure) when subjected to dehydration.

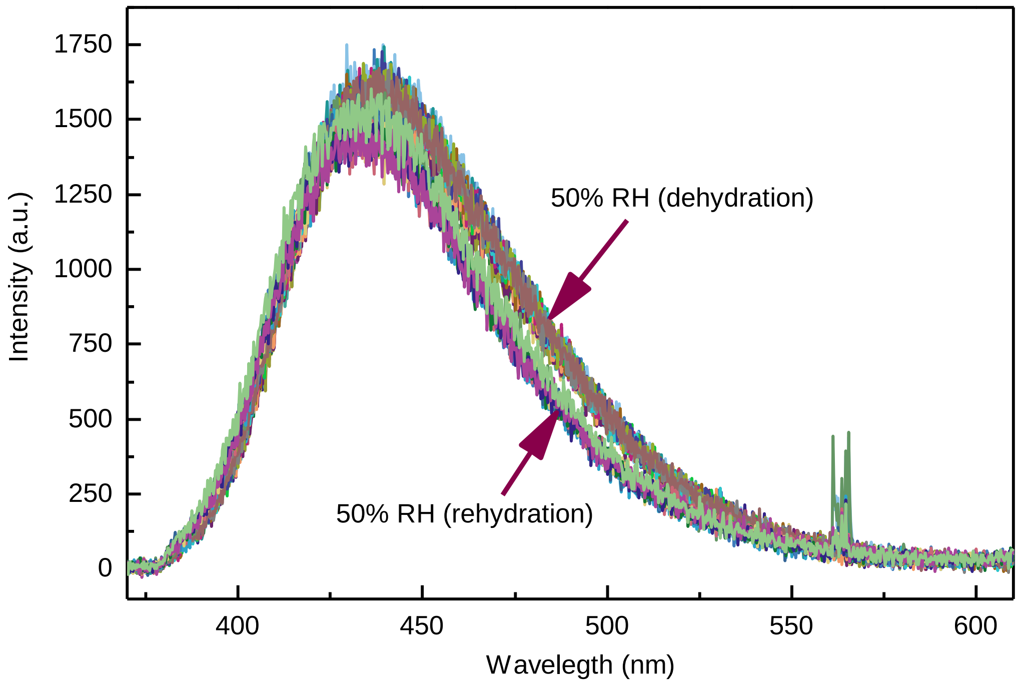

Figure S3. The fluorescence spectra of Laurdan embedded in an exemplary one-component solid supported lipid bilayer composed of di14:1-Δ9cis-PC lipids collected for ~50% relative humidity. The spectra originate from 30 distinct spots within the sample, separated by a distance ranging from a few to thousands of micrometers (the diameter of the mica flake is 8 mm). 20 spectra were collected at 50% RH during the dehydration and 10 during the rehydration process.

Note 2.

As a control, we measured the fluorescence spectrum of Laurdan layer deposited on a solid support in extreme hydration conditions: dried and exposed to bulk water. In the first place, we verified the fluorescence spectrum of Laurdan in solvents of distinct polarity, for which the Laurdan spectrum is known – chloroform and methanol. When dissolved in chloroform, Laurdan emission exhibits the maximum intensity at ~433 nm and in methanol at ~494 nm (see Fig. S4b), which is consistent with the literature.[2] After drying from both organic solvents, the fluorescence spectrum of Laurdan layer is blue-shifted with a maximum intensity centered at ~415-420 nm (see Fig. S4b,c), which is most likely a manifestation of the locally excited state.[3] We note that the deposition of Laurdan on solid support yields a formation of the isolated islands of aggregated and/or crystalline form of the probe (see Fig. S4a).

Intriguingly, exposure of such crystals/aggregates to bulk water neither shifts the spectrum nor changes its shape (see Fig. S4b). It is in accordance with the previously reported data demonstrating that Laurdan exhibits fluorescence emission maximum at around 410-415 nm in water.[4] In another study on extensive photophysical characterization of Laurdan and its derivatives, Laurdan fluorescence in water was not detected at all.[5] However, its derivative with an additional carboxymethyl group (MoC-Laurdan) was found to fluoresce in water with a very marked blue shift (maximum centered at around 410 nm), which was attributed to the fluorescence of non-hydrated aggregates. Whereas C-Laurdan (with one methyl group less with respect to Laurdan), which is highly soluble in water undergoes the expected red-shift and its fluorescence emission maximum is centered at ~520 nm.

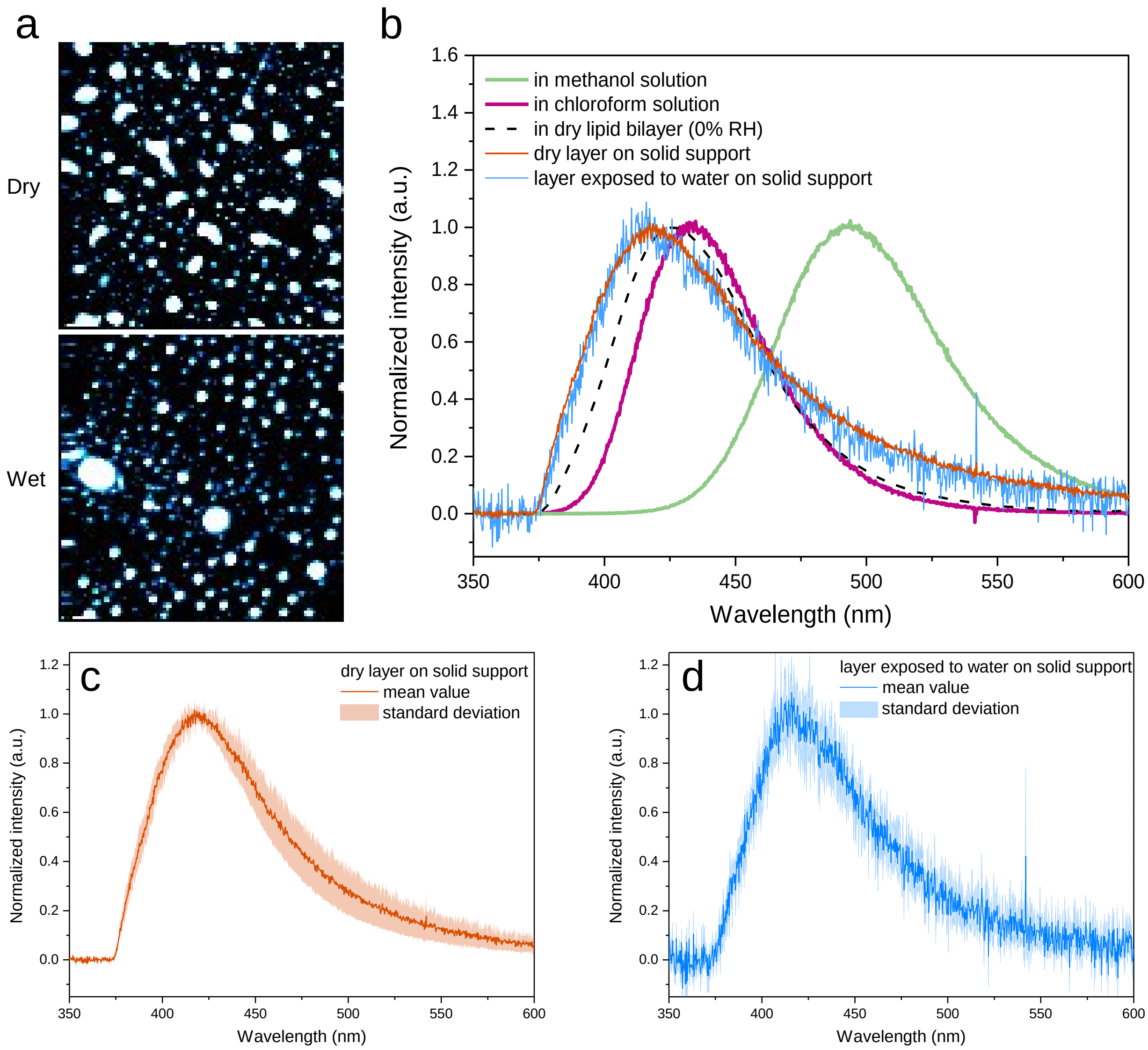

Figure S4. (a) Fluorescence microscopy images of Laurdan deposited on clean glass coverslip after drying from ~1 µM chloroform solution (Dry) and after exposing it to bulk ultrapure water (Wet). Images reflect the sample area of dimensions 50 x 50 µm. Contrast was adjusted and color was added for better visualization. (b) Fluorescence spectra of Laurdan on glass coverslip in dry and wet conditions averaged over at least 9 spectra collected from distinct spots within the sample area presented on panel *a* as well as of Laurdan in methanol and chloroform solutions. For the reference, the fluorescence spectrum of Laurdan in dry (0% RH) solid-supported lipid bilayer composed of di14:1-Δ9cis-PC is presented. Fluorescence spectra of Laurdan on glass coverslip in dry (c) and wet (d) conditions presented on panel *c* with added uncertainties are standard deviations, denoted as shadows around mean values. Spectra were normalized to better visualize the differences.

Noteworthy, we evaluated whether the fluorescence spectrum of the dried Laurdan layer exposed to pure water changes with time, but even after 17 hours, no changes were detected. Our results along with the available literature point in the direction that water molecules do not permeate the Laurdan layer, which most likely consist of tightly packed aggregates/crystals and that the intermolecular interactions between Laurdan molecules dominate over interactions with the interfacial water that would decrease the emitted energy by dipolar relaxation.

Regardless of the hydration condition (dry or wet), minor differences in the shape of the emission band between distinct spots within the sample was observed, but no clear trend regarding for instance the size of the crystal/aggregate or whether the spectrum was collected from in between the isolated

islands was found. To visualize the extent of the variability of the fluorescence spectra shape, in Figure S4c and S4d we plotted uncertainties (standard deviations), denoted as shadows around mean values. We infer that the Laurdan fluorescence spectrum depends on the state of aggregation and or whether it is in a crystalline form. Compared to its emission in a dried lipid membrane, it is blue-shifted by 5-10 nm (see Figure S4b, dashed black line). It must be emphasized however, that Laurdan in lipid membrane is expected to exist in its monomeric form, while on the support we deal with crystals and/or aggregates, for which probe-probe intermolecular interactions such as van der Waals forces and π-π interactions have to be taken into account.

More noise is evident to the normalized averaged spectrum of Laurdan layer exposed to bulk water because in wet conditions the overall fluorescence intensity decreased when compared to dry conditions, possible because of the collisional quenching with water molecules (see Fig. S4b-d).

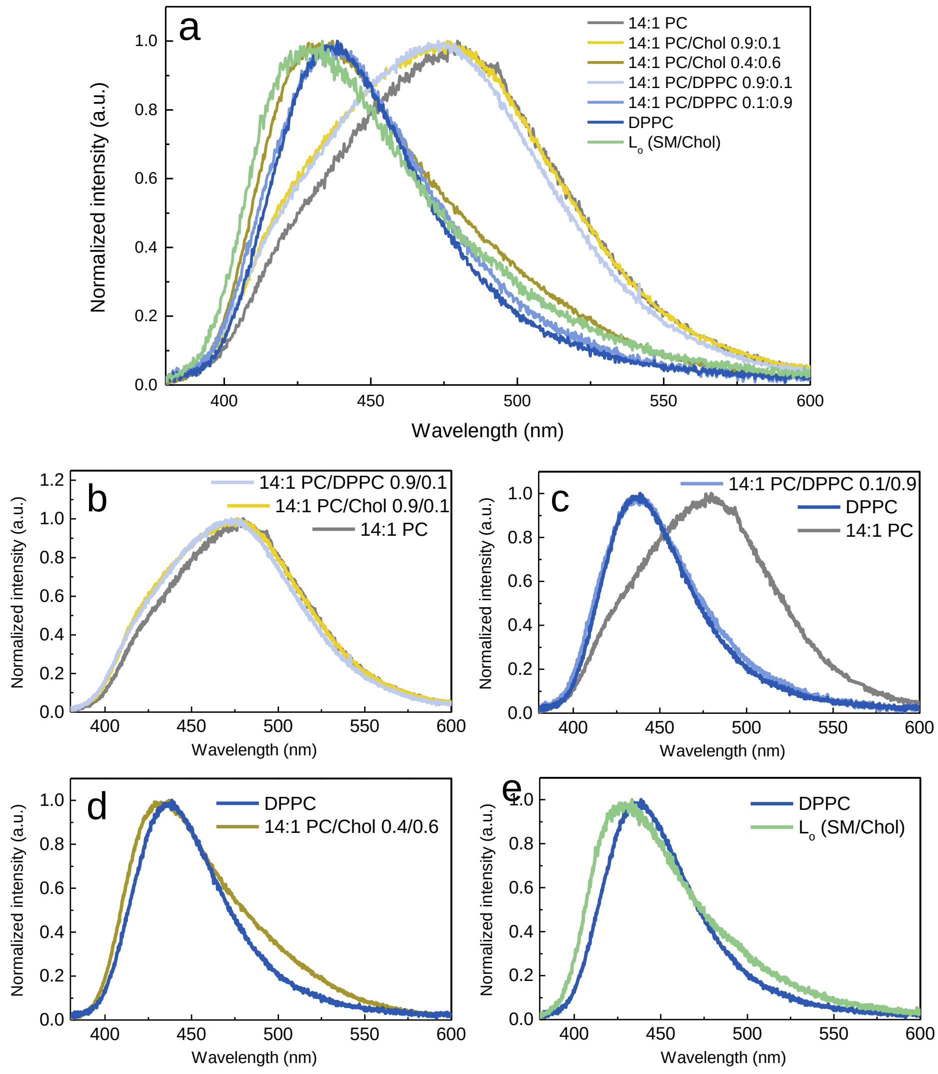

Figure S5. Fluorescence spectra of Laurdan embedded in exemplary solid-supported lipid bilayers composed of pure DPPC and binary mixtures of di14:1-Δ9*cis*-PC and DPPC at x_{DPPC} = 0.1 and 0.9 (blue shades, panels *a-e*). For each membrane composition the spectrum is averaged from 30 background-corrected spectra collected from distinct spots within the sample. For the comparison, exemplary fluorescence spectra of Laurdan in solid-supported lipid bilayers composed of pure di14:1-Δ9cis-PC (gray, panels *a-c*) as well as its mixtures with cholesterol at molar fractions x_{Chol} = 0.1 (yellow shade, panel *a*) and 0.6 (yellow shade, panels *a* and *d*) and in liquid-ordered phase (L_o) from the phase-separated membrane (green, panels *a* and *e*) are presented. Spectra were normalized to better visualize the differences.

Note 3.

At a very low molar fraction of DPPC (x_{DPPC} = 0.1), the Laurdan fluorescence spectrum is slightly blue-shifted with respect to pure di14:1-Δ9cis-PC bilayer (Fig. S5b), similarly as for x_{Chol} = 0.1, indicative of a minor increase of the membrane rigidity in both cases. At very high molar fractions of DPPC, namely x_{DPPC} = 0.9 and 1.0, the Laurdan fluorescence spectrum is prominently blue-shifted, as expected for the gel phase (Fig. S5c). Molecular movements in the gel phase are relatively constrained due to the high degree of order and packing of the lipid molecules, therefore dipolar relaxation around the Laurdan probe is strongly hampered. The possibility that the local environment of the Laurdan probe in gel-phase DPPC bilayer might be characterized by slightly decreased polarity with respect to the di14:1-Δ9cis-PC bilayer, due to reduced water penetration down to lipid carbonyl level, also cannot be ruled out. No significant differences in the fluorescence spectrum can be observed between pure DPPC and di14:1-Δ9cis-PC/DPPC 0.1/0.9 bilayers, although for the bilayer with very low molar fraction of di14:1-Δ9cis-PC, the spectrum seems to be a bit broader, which can be a manifestation of a disrupted tight gel-phase packing of DPPC introducing a broader distribution of Laurdan's local environments (Fig. S5c). When the fluorescence spectrum of Laurdan in DPPC matrix is compared to the di14:1-Δ9cis-PC bilayer with cholesterol at molar fraction x_{Chol} = 0.6 (Fig. S5d), it can be seen that for the sample with cholesterol, the spectrum is slightly blue-shifted (by ~5 nm) but at the same time has a higher contribution of a long-wavelength band associated with Laurdan within a readily relaxing environment. Given almost half of the concentration of cholesterol when compared to DPPC and the observed changes, it is clear that cholesterol has a stronger effect on the Laurdan's spectral response than a saturated phospholipid such as DPPC. This highlights further the unique character of this sterol molecule. In the figure above we presented also Laurdan's spectrum in L_o phase from the phase-separated SLB. Interestingly, when the newly acquired data are apposed to the probe's fluorescence spectrum in L_o phase (Fig. S5e), it is clear that although both phases – gel (DPPC) and L_o (SM/Chol) are considered rigid, Laurdan can distinguish them. It is in accordance with the results presented in the previous study employing different environment-sensitive probes demonstrating that gel and L_o phases exhibit similar fluidity but the latter is less hydrated.[6]

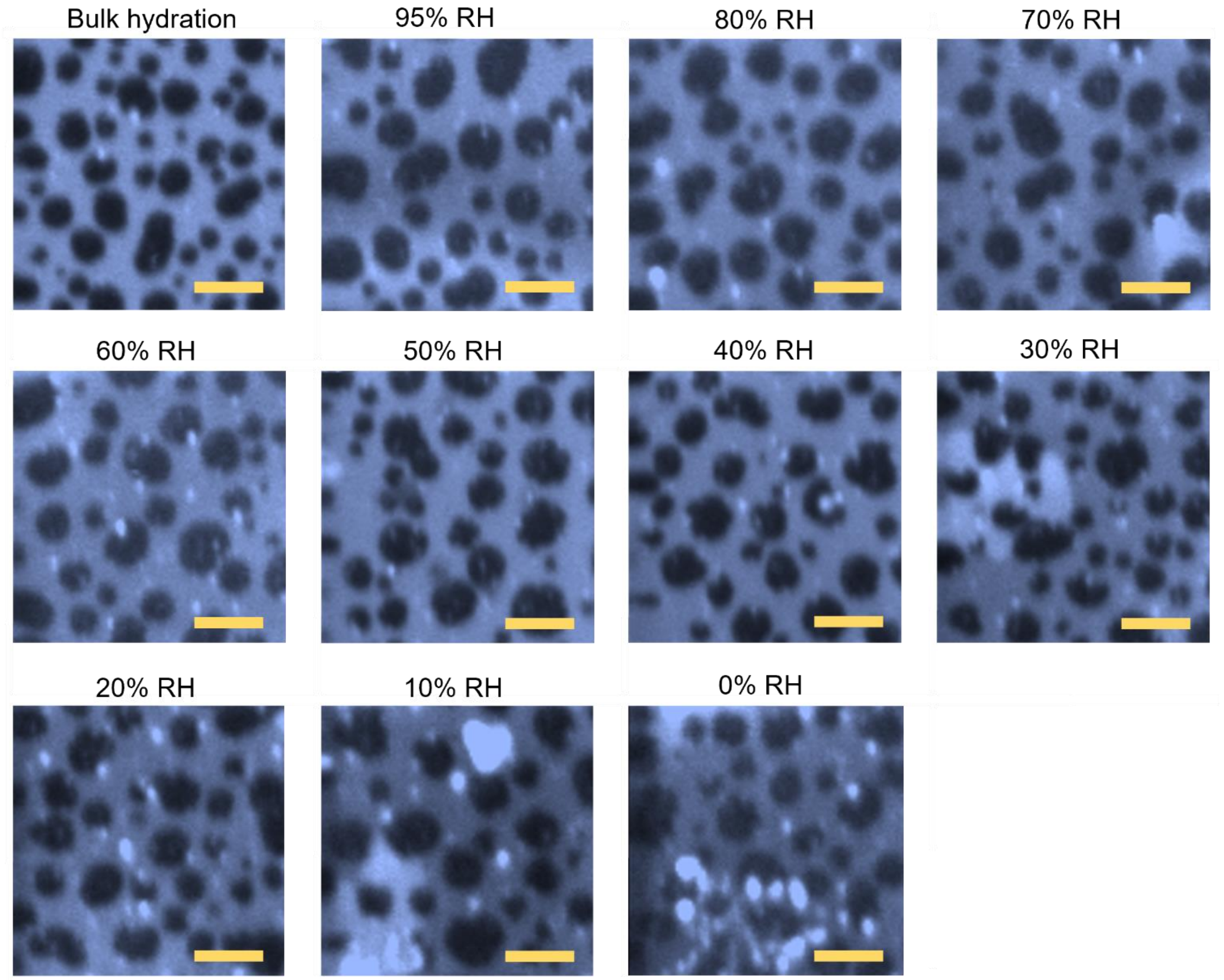

Figure S6. Fluorescence microscopy images of an exemplary phase-separated solid-supported lipid bilayer composed of an equimolar mixture of di14:1-Δ9*cis*-PC, cholesterol, and egg sphingomyelin as a function of membrane hydration state. The membrane exhibits phase separation into liquid-disordered (bright regions) and liquid-ordered (dark regions) domains. The membrane was labeled with Laurdan, which distributes evenly in the membrane regardless of phase, and Atto 633-DOPE, which localizes mainly in the liquid-disordered phase, and from this comes the contrast. The concentration of each dye was 0.1% mol. Each image represents a different area within the sample. The images shown originate from one of the four phase-separated samples analyzed in this work. The scale bar corresponds to 5 μm.

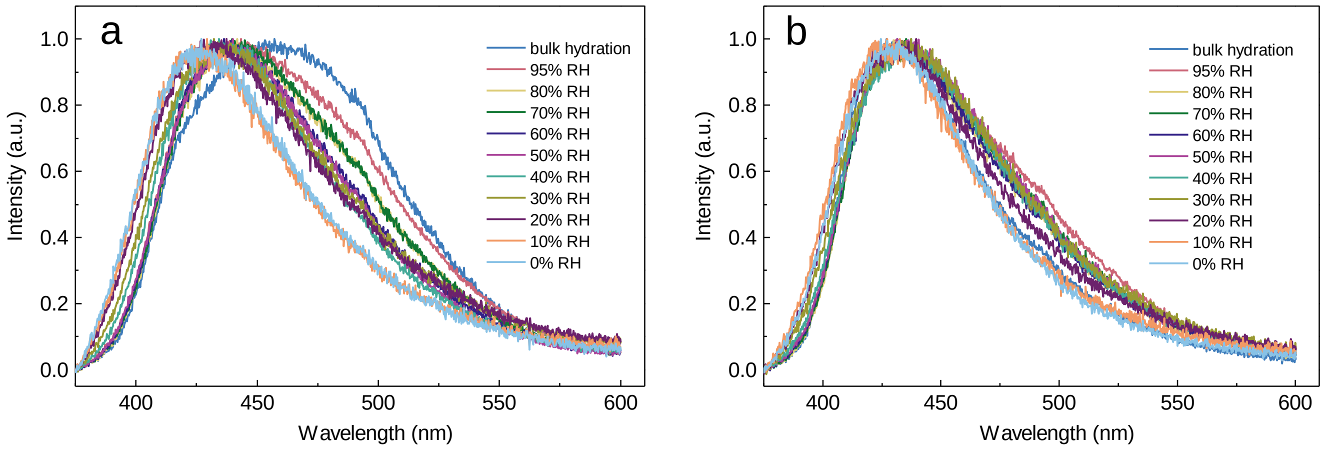

Figure S7. Fluorescence spectrum of Laurdan embedded in an exemplary phase-separated solid supported lipid bilayer composed of an equimolar mixture of di14:1-Δ9*cis*-PC, cholesterol, and egg sphingomyelin as a function of membrane hydration state collected from (a) liquid-disordered domains and (b) liquid-ordered domains. The spectra shown on both panels originate from one of the four phase-separated samples analyzed in this work (same sample as in Fig. S6). For each hydration state and each phase, the spectrum is averaged from at least 10 background-corrected spectra collected from distinct domains within the 20 x 20 μm sample area. The resulting spectra were normalized to better visualize the changes.

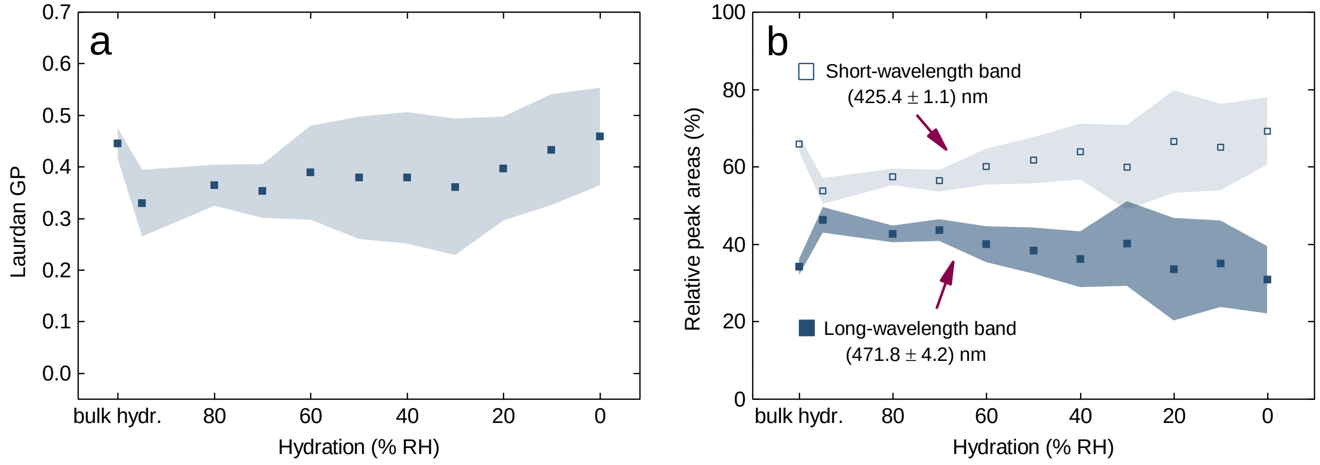

Figure S8. (a) Laurdan GP as a function of hydration level of liquid-ordered domains from phase-separated solid supported lipid bilayer composed of an equimolar mixture of di14:1-Δ9*cis*-PC, cholesterol, and egg sphingomyelin. (b) The relative area of the two log-normal functions that give the best fit to the Laurdan emission spectra in the same SLB system as a function of membrane hydration state. Open and full symbols are used for short- and long-wavelength bands, respectively. The data shown on both panels are averaged over four different samples. The uncertainties are standard deviations, denoted as shadows around mean values.

Note 4.

The insensitivity of Laurdan's emission spectrum in the liquid-ordered phase to changes in hydration over a fairly wide range is unlikely to indicate that no changes are occurring within this phase, but rather that Laurdan is not the appropriate dye to probe them. The liquid-ordered phase composed of saturated phospholipids (egg sphingomyelin in our case) and a high proportion of cholesterol is found to be the least hydrated and the most ordered among all the possible phases.[6,7] It is also reflected in the log-normal decomposition results (Fig. S7b), which revealed the considerably low contribution of long-

wavelength band in the Laurdan emission even at fully hydrated conditions compared to liquid-disordered domains or pure phospholipid bilayer. Even if the functional groups of sphingomyelin at the membrane depth where Laurdan resides are partially hydrated, its dipolar relaxation is slow compared to Laurdan's fluorescence timescale.[8] The stiff lipid acyl chains implicate the negligible contribution of Laurdan population surrounded by the relaxable environment to the emission spectrum over a wide range of membrane hydration states. Hence, in the main text we focused on the changes occurring within the liquid-disordered phase, for which the rate of dipolar relaxation is comparable to Laurdan fluorescence lifetime.[8]

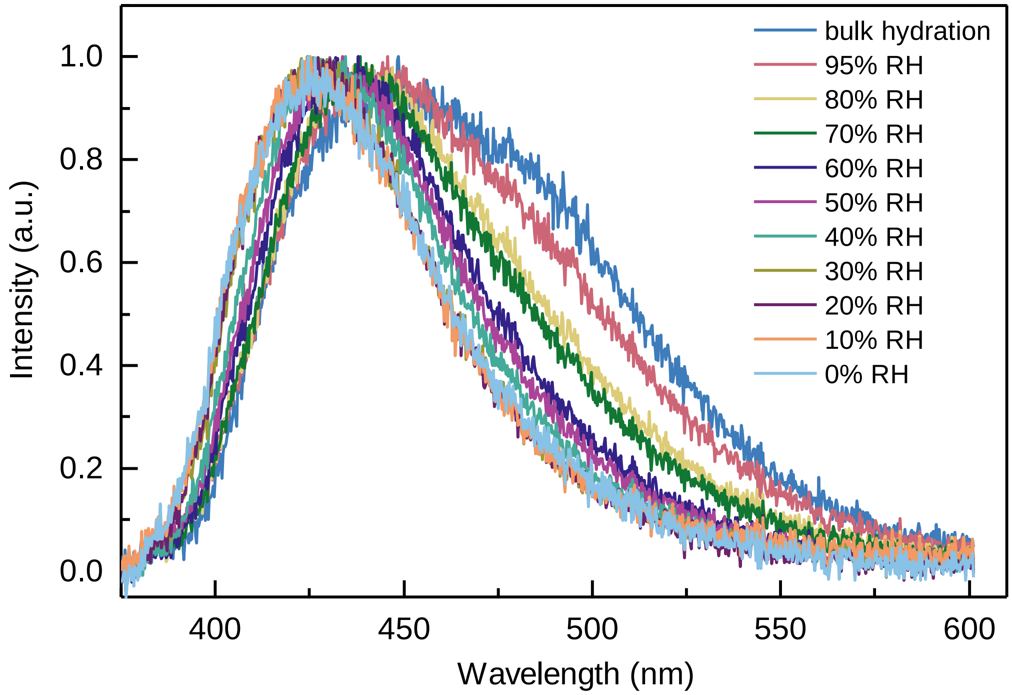

Figure S9. Fluorescence spectrum of Laurdan embedded in an exemplary solid-supported lipid bilayer composed of a binary mixture of di14:1-Δ9*cis*-PC and cholesterol at x_{Chol} = 0.3 as a function of membrane hydration state. For each hydration state, the spectrum is averaged from at least 10 background-corrected spectra collected from distinct spots within the 20 x 20 μm sample area. The resulting spectra were normalized to better visualize the changes.

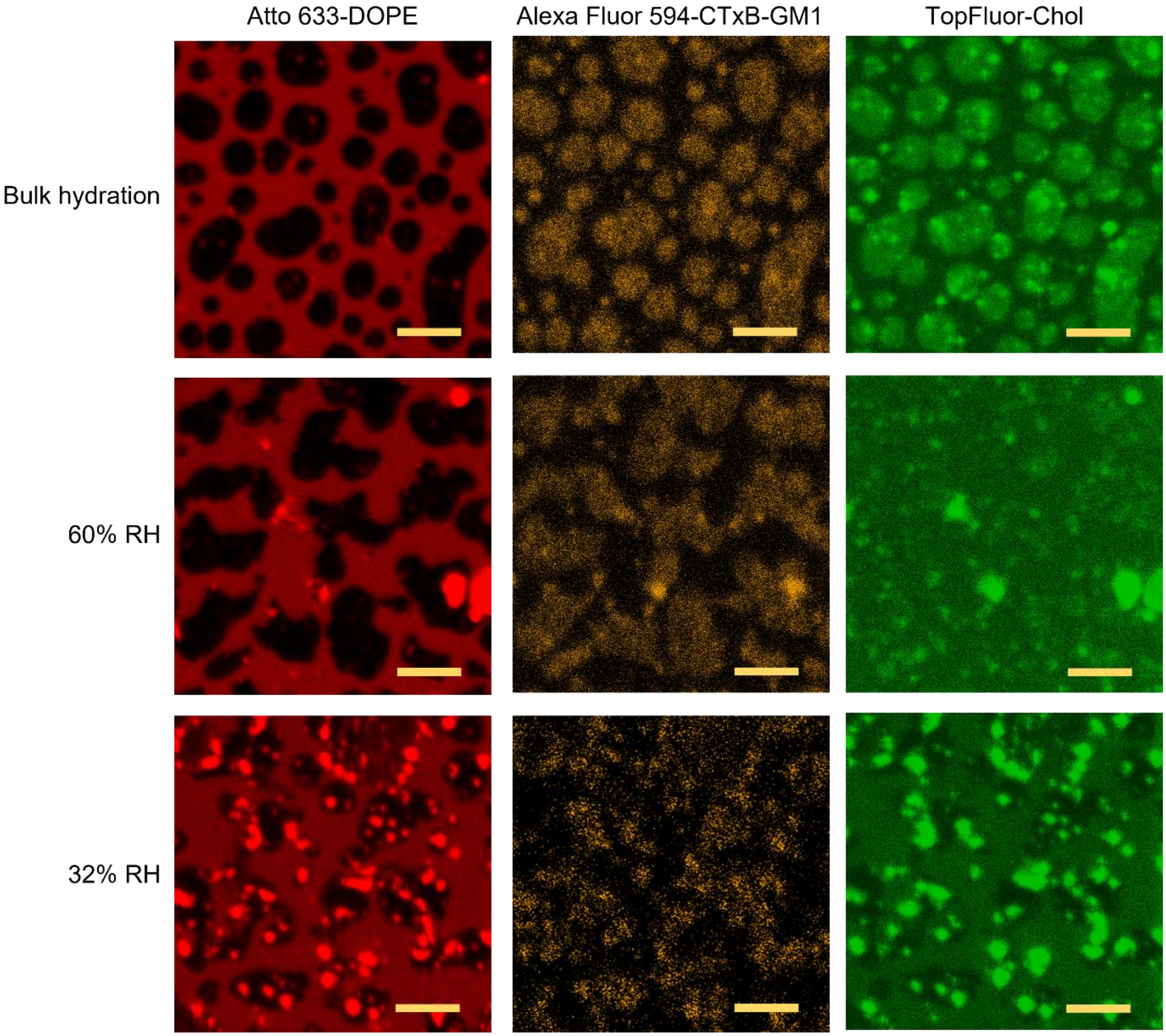

Figure S10. Confocal fluorescence microscopy images of a triple-labeled phase-separated solid-supported lipid bilayer composed of an equimolar mixture of di14:1-Δ9*cis*-PC, cholesterol, and egg sphingomyelin for three different membrane hydration states. Liquid-disordered phase is labeled with Atto 633-DOPE (red, left column), liquid-ordered phase is labeled with Alexa Fluor 594-CTxB-GM1 complex (yellow, middle column) and cholesterol, which partitions in both phases is labeled with TopFluor-Chol probe (green, right column). Images for each hydration state originate from different sample area. Under fully hydrated conditions higher intensity of TopFluor-Chol is found in liquid-ordered domains, denoting that liquid-ordered phase contains more cholesterol than liquid-disordered phase, in accordance with the previous reports. At 60% RH the contrast virtually vanishes, indicative of homogenous distribution of cholesterol between distinct phases. At 32% RH the contrast is reversed with respect to the fully hydrated conditions, denoting that at low hydration conditions liquid-disordered phase contains more cholesterol than liquid-ordered phase. The concentration of each dye was 0.1% mol. The scale bar corresponds to 5 μm.

References

(1) Chattopadhyay, M.; Krok, E.; Orlikowska, H.; Schwille, P.; Franquelim, H. G.; Piatkowski, L. Hydration Layer of Only a Few Molecules Controls Lipid Mobility in Biomimetic Membranes. *J. Am. Chem. Soc.* **2021**, *143* (36), 14551–14562.

(2) Hornum, M.; Kongsted, J.; Reinholdt, P. Computational and Photophysical Characterization of a Laurdan Malononitrile Derivative. *Phys. Chem. Chem. Phys.* **2021**, *23* (15), 9139–9146.

(3) Owen, D. M.; Rentero, C.; Magenau, A.; Abu-Siniyeh, A.; Gaus, K. Quantitative Imaging of Membrane Lipid Order in Cells and Organisms. *Nat. Protoc. 2012 71* **2011**, *7* (1), 24–35.

(4) Hu, S.; Yao, Z.; Ma, X.; Yue, L.; Chen, L.; Liu, R.; Wang, P.; Li, H.; Zhang, S. T.; Yao, D.; Cui, T.; Zou, B.; Zou, G. Pressure-Induced Local Excitation Promotion: New Route toward High-Efficiency Aggregate Emission Based on Multimer Excited State Modulation. *J. Phys. Chem. Lett.* **2022**, *13* (5), 1290–1299.

(5) Mazeres, S.; Joly, E.; Lopez, A.; Tardin, C. Characterization of M-Laurdan, a Versatile Probe to Explore Order in Lipid Membranes. *F1000Research* **2014**, *3*, 172.

(6) M'Baye, G.; Mély, Y.; Duportail, G.; Klymchenko, A. S. Liquid Ordered and Gel Phases of Lipid Bilayers: Fluorescent Probes Reveal Close Fluidity but Different Hydration. *Biophys. J.* **2008**, *95* (3), 1217–1225.

(7) Warschawski, D. E.; Devaux, P. F. Order Parameters of Unsaturated Phospholipids in Membranes and the Effect of Cholesterol: A 1H-13C Solid-State NMR Study at Natural Abundance. *Eur. Biophys. J.* **2005**, *34* (8), 987–996.

(8) Leung, S. S. W.; Brewer, J.; Bagatolli, L. A.; Thewalt, J. L. Measuring Molecular Order for Lipid Membrane Phase Studies: Linear Relationship between Laurdan Generalized Polarization and Deuterium NMR Order Parameter. *Biochim. Biophys. Acta - Biomembr.* **2019**, *1861* (12), 183053.
